# Coordinated clinical trial networks in nephrology: strengthening research infrastructure and improving trial delivery

**DOI:** 10.1093/ckj/sfag257

**Published:** 2026-08-06

**Authors:** Martin H de Borst, Aaltje Y Adema, Jeetindra Balak, Hiddo J L Heerspink, Melanie Schoutteten, Ellen K Hoogeveen, Ewout J Hoorn, Niki Leenders, Sabine C A Meijvis, Gurbey Ocak, Joris I Rotmans, Femke Waanders, NEFRO-NL Collaborators, M de Borst, J Rotmans, L Vogt, E Hoorn, S Meijvis, R Duivenvoorden, N Leenders, G Ocak, E Hoogeveen, F Waanders, A Adema, M Eijgelsheim, J van der Leeuw, A Kramer, N Cnossen, W Bax, J vander Net, M van Buren, C de Roij van Zuijdewijn, P Luik, P Rootjes, L Siddiqi, M Schoutteten, J-J Snoep, S Logtenberg, Raphael Duivenvoorden, Liffert Vogt

**Affiliations:** Departments of Internal Medicine, Division of Nephrology and Clinical Pharmacology, University Medical Center Groningen, University of Groningen, Groningen, The Netherlands; Department of Internal Medicine, Frisius Medical Center Leeuwarden, The Netherlands; Department of Internal Medicine, Haga Hospital, The Hague, The Netherlands; Departments of Nephrology and Clinical Epidemiology, Leiden University Medical Center, Leiden, The Netherlands; Departments of Clinical Pharmacology, University Medical Center Groningen, University of Groningen, Groningen, The Netherlands; Department of Internal Medicine, Catharina Hospital, Eindhoven, The Netherlands; Department of Internal Medicine, Jeroen Bosch Hospital, Den Bosch, The Netherlands; Department of Clinical Epidemiology, Leiden University Medical Center, Leiden, The Netherlands; Department of Internal Medicine, Division of Nephrology and Hypertension, Erasmus Medical Center, Rotterdam, The Netherlands; Department of Nephrology, Maastricht University Medical Center+, Maastricht, The Netherlands; Department of Nephrology, University Medical Center Utrecht, Utrecht, The Netherlands; Department of Internal Medicine, St Antonius Hospital, Nieuwegein, The Netherlands; Departments of Nephrology and Clinical Epidemiology, Leiden University Medical Center, Leiden, The Netherlands; Department of Internal Medicine, Isala Hospital, Zwolle, The Netherlands; Department of Nephrology, Radboud University Medical Center, Nijmegen, The Netherlands; Department of Nephrology, Amsterdam University Medical Center, Amsterdam, The Netherlands

**Keywords:** CKD, clinical trial, patient recruitment, research infrastructure, trial networks

## Abstract

Chronic kidney disease (CKD) affects ∼10% of the global population and is a leading cause of morbidity and mortality. Although therapeutic options in nephrology are rapidly expanding, the capacity to conduct large-scale clinical trials in Europe is increasingly constrained by fragmented infrastructure, complex administrative processes, and unequal access to trial participation. Coordinated clinical trial networks have emerged as a strategy to address these challenges. By enabling central feasibility assessment, harmonised contracting through pre-existing master agreements, and systematic outreach to all participating sites, such networks can accelerate trial start-up, improve recruitment, and enhance inclusivity. Nephrology Research Organisation The Netherlands (NEFRO-NL) represents a recent example of this approach. By linking hospitals nationwide, this trial network facilitates multicentre collaboration, supports investigator-initiated research, and promotes more efficient use of patient populations. While long-term impact on trial performance remains to be established, early experience suggests that coordinated trial networks can enhance efficiency and broaden participation in nephrology research. More broadly, coordinated clinical trial networks are likely to become essential infrastructure for delivering timely, high-quality, and inclusive nephrology research.

KEY LEARNING POINTS **What was known:**

- Clinical trial activity in nephrology is increasing, but trial execution in Europe is hindered by fragmented infrastructure, complex regulatory processes, and unequal access across hospitals.
- Patient recruitment is often slow, and many centres lack the infrastructure and expertise required for modern clinical trials.
- Coordinated trial networks have been proposed as a strategy to address these challenges, but their role in nephrology remains underexplored.

**This study adds:**

- We provide a conceptual framework describing how coordinated clinical trial networks can improve trial initiation, recruitment, and inclusivity in nephrology.
- Using the NEFRO-NL network as a case example, we illustrate how central feasibility assessment, master agreements, and coordinated site engagement can accelerate trial delivery.
- We place these findings in an international context, highlighting how similar models have strengthened trial capacity in other countries.

**Potential impact:**

- Coordinated trial networks may substantially improve efficiency and inclusivity of clinical trials, enabling faster translation of therapeutic advances into patient care.
- Adoption of network-based infrastructure could strengthen Europe’s position in the global clinical trial landscape.
- These models may support investigator-initiated research and facilitate large-scale national and international collaboration in nephrology.

Chronic kidney disease (CKD) affects one in seven individuals globally [1]. Within Europe, CKD prevalence averages 11%, which is slightly above the global median [2]. Although early CKD is asymptomatic, advanced CKD has a profound impact on life expectation, quality of life and participation in society. CKD is now considered one of the leading causes of death worldwide, most prominently driven by a strong and gradually elevated risk of cardiovascular disease as kidney function declines [3]. In 2023, globally 1.5 million people died from CKD; in addition, 11.5% of global deaths due to cardiovascular disease is attributable to CKD [1]. CKD-related mortality rate increased by 48% between 1990 and 2023, and predictions suggest that it will become the fifth highest cause of years of life lost globally by 2040 [1, 4]. The financial impact of CKD, and particularly kidney failure, on society is immense. In Europe, annual CKD-related healthcare costs are estimated at €140 billion [5]; annual healthcare-related costs per kidney failure patient at €44 000 [6] and additional costs due to loss of productivity at €23 000 [7]. Of note, the burden of chronic kidney disease is highest among historically disadvantaged populations who often have limited access to optimal medical therapies. This limited access importantly contributes to socioeconomic disparities in health outcomes in these patient groups [8].

## NEPHROLOGY TRIAL LANDSCAPE IS DEVELOPING RAPIDLY

Despite the major adverse impact of CKD on both the individual patient and global society, the overall number and quality of published randomized controlled clinical trials (RCTs) in nephrology have been remarkably low in comparison with other fields [9, 10]. In addition, patients with CKD have been structurally excluded from cardiovascular RCTs to a larger extent than expected on safety grounds, leading to underrepresentation in such trials over the past two decades [11]. Recently, however, this has changed. Over the past decade, several drugs with specific kidney and cardiovascular protective effects, including sodium glucose co-transporter 2 inhibitors, the non-steroidal mineralocorticoid receptor antagonist finerenone and glucagon-like peptide-1 receptor agonists, have been investigated in large clinical trials in CKD populations and with dedicated assessments of both kidney and cardiovascular outcomes [12–15, 16]. Furthermore, an array of new compounds that target deregulations of the immune system, including the complement system and immunoregulatory cytokines B-cell Activating Factor and A Proliferation-Inducing Ligand are now under investigation in glomerular diseases [17, 18]. Several pharmaceutical companies have new drugs in their pipelines that target the cardiovascular-kidney-metabolic syndrome, and are simultaneously tested in patients with metabolic (obesity, diabetes), cardiovascular (heart failure), or kidney disease or combinations of these [19]. Finally, emerging and innovative trial designs, including hierarchical composite endpoints and platform trials are now being adopted, opening new avenues for patient-oriented research in nephrology [20]. Together, these developments have led to a rapid increase in the number, complexity, and scale of nephrology trials. However, existing trial infrastructure has not kept pace with these developments, resulting in important barriers to trial initiation and execution. Here, we describe a conceptual framework for how coordinated trial networks can address these barriers, using the recently established Dutch trial network NEFRO-NL as a case example.

## EUROPEAN TRIAL INFRASTRUCTURE IS FACING CHALLENGES

The exciting recent developments provide major opportunities for scientific breakthroughs and improved patient care. However, the (Western) European trial infrastructure is facing challenges that threaten its position in the global clinical trial arena (Table 1). Europe is already losing global share, particularly to Asia and other regions, falling from 25% to 19% in 10 years [21]. Clinical trial execution in Europe has become much more time-consuming and expensive for several reasons. First, confidentiality agreements and clinical trial agreements between sponsors and participating hospitals can take months due to heterogeneous and complex administrative processes and variable policies. Second, obtaining ethical approval in Europe remains a complex and slow process that is still suffering from local variation despite the EU Clinical Trials Regulation. Third, the potential of patient resources is underexploited because of unequal access between hospitals. While university medical centres often have more direct access to clinical trials due to established connections with the pharmaceutical industry and visibility in the scientific field, larger non-academic hospitals may have access to substantial patient populations, particularly for more common diseases, but have less access to clinical trials. Finally, not all hospitals have the infrastructure and expertise to participate in contemporary clinical trials that are highly regulated and specialized. This restricts trial capacity, particularly when collaboration between hospitals is limited. Thus, RCTs in Western countries face slower patient recruitment. This is a challenge that extends beyond Europe as it also affects the United States, likely reflecting increasing trial complexity as well as difficulties in finding suitable patients [21]. At the same time, limited access to trials and highly specific criteria likely affect representativeness of trial populations for actual clinical practice populations, for example regarding sex and ethnic background.

**Table 1:** Challenges in clinical trial management and solutions provided by trial networks.

Challenge	Consequence	Solutions provided by trial networks
Highly variable and complex contracting requirements across sites	Prolonged and unpredictable study start-up timelines	Master agreements between hospitals and the network, and between the network and sponsors, enabling rapid contracting
Complex and time-consuming ethical approval procedures with local variation	Delays in study initiation and duplication of efforts across sites	Standardized protocols and centralized coordination of ethics submissions to streamline approval processes
Fragmented trial access across hospitals	Underutilization of patient populations and reduced representativeness of trial cohorts	Transparent and coordinated site selection procedures ensuring that all participating hospitals can be considered for each study
Sequential site activation due to site-specific contracting and feasibility processes	Delayed trial initiation and slow patient recruitment	Central feasibility assessment and pre-existing master agreements enabling faster site activation
Limited infrastructure and expertise in smaller or non-academic centres	Reduced participation of these centres in clinical trials and missed recruitment opportunities	Shared infrastructure, training, and knowledge exchange within the network to build capacity across sites
Unequal patient access to clinical trials	Reduced inclusivity and potential bias in trial populations	Network-wide approach to site engagement, ensuring systematic outreach to all participating hospitals and broader patient access
Increasing complexity of trial designs and regulatory requirements	Reduced feasibility of investigator-initiated studies	Central trial support, including methodological expertise and coordination within the network

## THE POTENTIAL OF TRIAL NETWORKS

**Figure 1:**
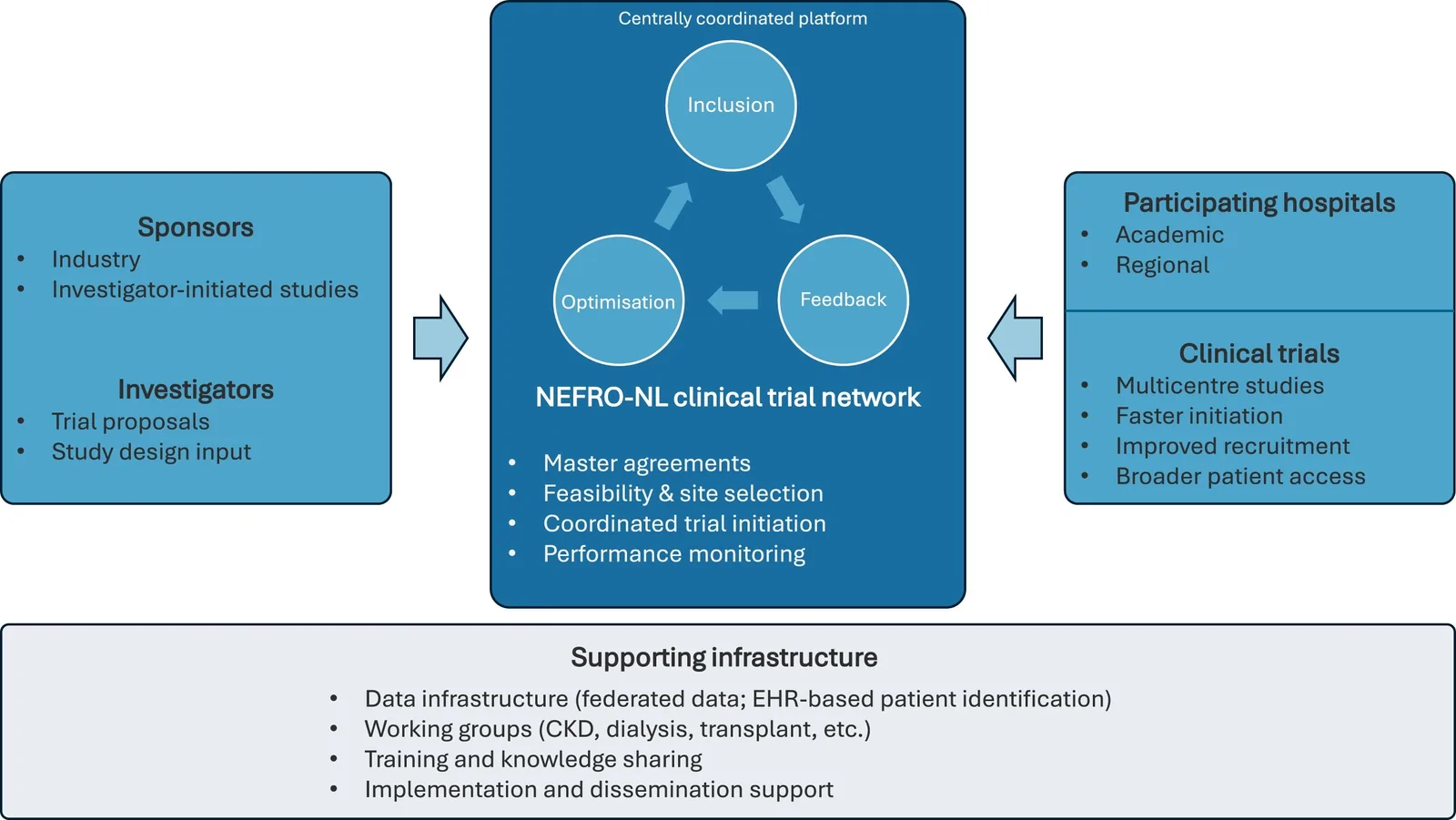
Structure and core functions of a national nephrology clinical trial network. The NEFRO-NL clinical trial network acts as a centrally coordinated platform connecting sponsors, investigators, and participating hospitals. Core functions include harmonised contracting through master agreements, central feasibility assessment and site selection, coordinated study start-up, and performance monitoring. Participating hospitals contribute to multicentre clinical trials, enabling faster initiation, improved recruitment, and broader patient access. Continuous feedback and optimisation cycles support ongoing improvement. Supporting infrastructure includes data systems enabling federated data use and electronic health record (EHR)-based patient identification, thematic working groups, and training and knowledge sharing.

In the light of the opportunities and challenges outlined above, new strategies must be adopted to reinforce the European position in the global trial landscape. Collaboration in trial networks provides the potential to improve trial initiation and enhance execution efficiency. First, trial networks can provide structural and overarching (master) arrangements between hospitals and the network, and between the network and sponsors (Fig. 1). Such master arrangements replace the need for individual contracts between parties for each study, reducing study preparation time by months. Second, using standardized protocols and centralized procedures, ethical committee application and clinical trial agreement procedures are streamlined, further accelerating trial initiation. Third, all hospitals within the network are offered the option to participate in trials. A standard operating procedure for site selection ensures balanced and fair adjudication of trials to centres, overseen by a site selection committee. Fourth, direct access to a large group of hospitals promotes patient recruitment, and collaborating in a network can leverage developments in (national) data infrastructure to improve access and facilitate faster patient identification and recruitment (see next paragraph). Finally, sharing infrastructure and knowledge helps all sites improve continuously (Fig. 1). Importantly, trial networks do not eliminate all regulatory and logistical challenges but substantially reduce inefficiencies and improve coordination across sites.

## TRIAL NETWORKS CAN BOOST NATIONAL DATA INFRASTRUCTURE

At a national level, the variability in electronic health record systems and their (currently) limited feasibility for research applications make it highly challenging to generate high-level data on patients meeting very specific trial criteria. In fact, very few hospitals in The Netherlands have solutions in place to adequately identify specific patient populations locally. Several problems hamper nationwide collaboration in patient identification, including inconsistent source registration, absent or poorly functioning connections between hospitals, strict privacy legislation and lengthy contract processes for data transfer. Case-by-case data collection is time-consuming and expensive, and therefore new solutions are required to facilitate larger-scale patient identification more cost-efficiently. Collaboration in a trial network could provide an impulse to reorganize research infrastructure at the national level. Federated approaches allow for local collection of data that is subsequently shared with the network at an aggregated level to avoid patient-level data transfer.

Another opportunity for research networks is to promote the secondary use of patient data, particularly from investigator-initiated trials. Secondary use of trial data and biobanked samples are enabled by adding such information to trial documentation including patient information files and informed consent forms. In the context of clinical trials, (re-)analysis of data originally collected for other purposes can yield powerful new conclusions. Additional potential applications include *de novo* observational studies using previously collected trial data and samples, and the inclusion of historical and real-world data in clinical trials. Trial networks likely also lead to centralization of existing datasets, which can be brought into the context of a national research infrastructure, enhancing access to research datasets for a broader scientific population.

## INITIATION OF A DUTCH CLINICAL TRIAL NETWORK

In February 2025, a clinical research network named Nephrology Research Organisation The Netherlands (NEFRO-NL) was established. The network was founded by 11 hospitals and was subsequently opened for all other hospitals in The Netherlands (total *N* = 70). In the subsequent year, the number of participating hospitals increased to 24. Together, these hospitals manage over 150 000 CKD patients. Within its first year, the network has already facilitated five multicentre trials (two investigator-initiated and three industry-sponsored), with seven more trials in preparation. While the long-term impact on trial performance remains to be established, early experience from NEFRO-NL suggests that coordinated trial infrastructure can accelerate trial initiation and recruitment at a national scale.

## KEY FEATURES OF THE NEFRO-NL MODEL

NEFRO-NL functions as a central portal connecting sponsors, investigators, and trial sites. It coordinates site selection, harmonises contracts, and supports study set-up, while leaving trial execution to the participating centres. This model enables:

- **Streamlined procedures**: A nationally agreed clinical trial agreement template [22] and centralised budget negotiations accelerate study start-up;
- **Rapid site response**: Participating hospitals commit to answering feasibility queries within two weeks;
- **Quality oversight**: Performance indicators across sites are monitored, fostering continuous improvement;
- **Working groups**: Thematic groups (glomerular disease, dialysis, transplantation, hereditary disorders, and chronic kidney disease) bring together key opinion leaders to evaluate study proposals, advise on trial design, and generate new investigator-led initiatives.

NEFRO-NL is supported through a combination of study-specific service agreements with sponsors, institutional contributions from participating hospitals, and external funding. The network operates in close collaboration with key stakeholders, including patient organisations, the Dutch Kidney Foundation, and the Dutch Federation of Nephrology, to ensure alignment with clinical priorities and patient needs.

**Figure 2:**
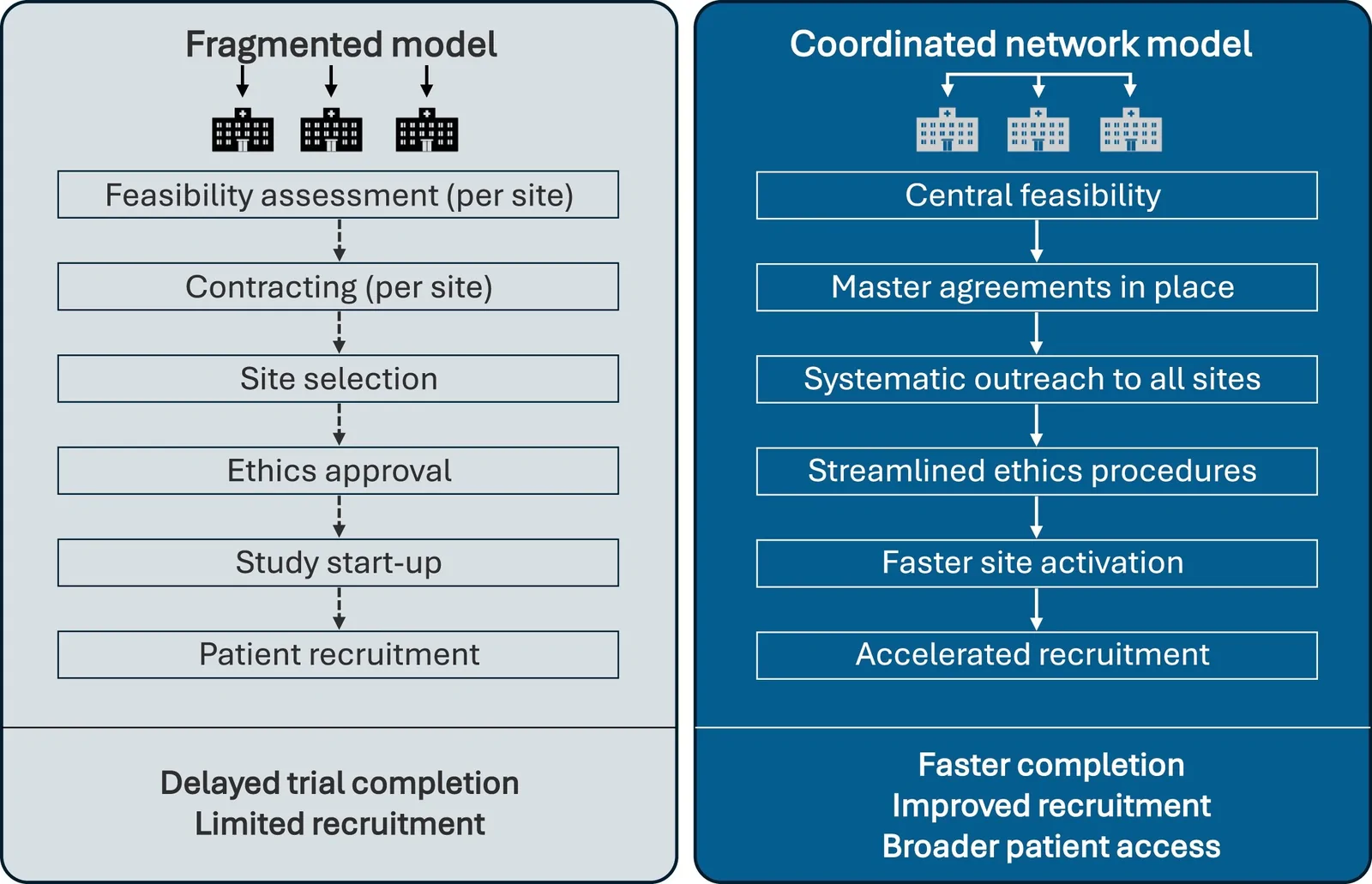
Impact of a coordinated clinical trial network on trial start-up and execution. In fragmented models (left), trial processes including feasibility assessment, contracting, site selection, and ethics approval are performed sequentially and independently across sites, resulting in delayed trial completion and limited recruitment. In a coordinated network model (right), central feasibility assessment, pre-existing master agreements, and systematic outreach to all sites enable faster site activation and broader participation. This leads to accelerated recruitment, improved inclusivity, and faster trial completion.

The impact of this coordinated approach on trial start-up and execution is illustrated in Fig. 2. The benefits for participating hospitals include greater exposure to high-quality trials, reduced administrative burden, and opportunities to contribute to both patient care and scientific discovery. These early experiences indicate that a coordinated infrastructure has the capacity to scale rapidly across a national healthcare system.

## LESSONS LEARNED FROM INTERNATIONAL NETWORKS

The design of NEFRO-NL is informed by the experience of established trial networks. The Australasian Kidney Trials Network (AKTN), founded in 2005, has demonstrated how collaborative infrastructure can change practice [23]. Landmark AKTN-led studies, such as CKD-FIX [24] and IDEAL [25] influenced global guidelines and set new standards for pragmatic nephrology research. Similar networks exist in other countries including the Can-SOLVE network in Canada and the United Kingdom Kidney Research Consortium (UKKRC). These nephrology research networks, as well as trial networks in other fields, have shown that continuous learning across sites increases recruitment efficiency and data quality, while also enhancing clinician engagement. The observed improvements in recruitment and trial efficiency are likely multifactorial, but coordinated infrastructure appears to be a key enabling factor.

A consistent finding across networks is that investigator-initiated research flourishes once a trial platform is in place. NEFRO-NL is explicitly designed to support such studies, by facilitating grant applications, streamlining multicentre coordination, and providing trial management services that small investigator groups cannot deliver alone. This structure is crucial in nephrology, where industry pipelines often focus narrowly on late-stage disease or commercially attractive therapies, leaving early-stage interventions, rare diseases, and health-service research underexplored. Future collaboration between national trial networks may enable truly international platform trials in nephrology.

## NATIONAL IMPACT AND INTERNATIONAL POTENTIAL

The Netherlands is well positioned to benefit from a national nephrology trial network. With its dense geography, strong academic climate, and existing culture of collaboration, a single platform can reach the majority of the country’s kidney patient population. This not only enhances the generalisability of study findings but also makes the Netherlands a more attractive partner for international trial consortia. Importantly, NEFRO-NL embeds continuous quality improvement into its structure. By monitoring inclusion rates, retention, and site performance, the network creates a feedback loop that improves each subsequent trial. This approach aligns with calls for learning health systems in nephrology, where clinical care and research are integrated to accelerate discovery and implementation.

## CHALLENGES AND OPPORTUNITIES AHEAD

The success of NEFRO-NL will depend on sustained participation, adequate funding, and maintaining the delicate balance between efficiency and scientific independence. Established successful networks have shown that long-term viability requires transparent governance, equitable distribution of trial opportunities, and clear value for both academic, non-academic and industry partners. As the network grows beyond its founding hospitals, ensuring inclusivity and avoiding concentration of trials in a few high-volume centres will be essential. At the same time, nationwide trial infrastructure such as NEFRO-NL provides major opportunities for investigator-initiated trials, including focused research priorities, improved trial quality, and patient involvement (Table 2). In addition, such infrastructure is fertile ground for young scientists through providing educational and career opportunities.

**Table 2:** Opportunities for investigator-initiated research enabled by clinical trial networks.

Opportunity	Impact	How trial networks enable this
Jointly established clinical research priorities	Better alignment of research with clinical needs and increased relevance of studies	Collaborative governance structures and thematic working groups define and prioritize research agendas
Educational and career opportunities for clinical scientists	Development of clinical research expertise and support for early-career investigators	Access to training, mentorship, and participation in multicentre trials within the network
Increased clinical trial quality	Improved study design, conduct, and data quality	Access to experienced biostatisticians, trial coordinators, monitors, and Good Clinical Practice training
Collaboration with patient representatives	Greater patient involvement and improved relevance and acceptability of studies	Structured engagement of patient representatives in study design and prioritization
Increased visibility and dissemination of research	Broader awareness and uptake of study findings	Shared communication platforms and joint publications across the network
Improved competitiveness for research funding	Higher success rates in grant applications	Demonstrated feasibility, larger recruitment potential, and coordinated infrastructure
Shared patient cohorts and data resources	Opportunities for observational studies and secondary analyses	Central coordination of datasets and facilitation of data sharing within regulatory frameworks
International collaboration with other trial networks	Ability to conduct large-scale, multinational trials	Alignment and collaboration with established networks in other countries (e.g. Australia, Canada, UK)

## CONCLUSION

The establishment of NEFRO-NL marks a major advance for Dutch nephrology research. By creating a unified trial infrastructure, the network promises faster, higher-quality, and more impactful studies. Experience from the AKTN and others suggests that such platforms catalyse investigator-initiated research, improve patient recruitment, and ultimately shape clinical practice. Although NEFRO-NL represents a national initiative, similar challenges and solutions are observed across multiple healthcare systems, suggesting that coordinated trial networks may represent a broadly applicable model. Coordinated trial networks are likely to become essential infrastructure for delivering high-quality, inclusive, and timely nephrology research in this era of rapidly expanding therapeutic opportunities in nephrology.

## Data Availability

There are no new data associated with this article.
